# Deep-learning system to improve the quality and efficiency of volumetric heart segmentation for breast cancer

**DOI:** 10.1038/s41746-021-00416-5

**Published:** 2021-03-05

**Authors:** Roman Zeleznik, Jakob Weiss, Jana Taron, Christian Guthier, Danielle S. Bitterman, Cindy Hancox, Benjamin H. Kann, Daniel W. Kim, Rinaa S. Punglia, Jeremy Bredfeldt, Borek Foldyna, Parastou Eslami, Michael T. Lu, Udo Hoffmann, Raymond Mak, Hugo J. W. L. Aerts

**Affiliations:** 1 Artificial Intelligence in Medicine (AIM) Program, Brigham and Women’s Hospital, Harvard Medical School, Boston, MA USA; 2 Department of Radiation Oncology, Dana-Farber Cancer Institute, Brigham and Women’s Hospital, Harvard Medical School, Boston, MA USA; 3 Cardiovascular Imaging Research Center, Massachusetts General Hospital, Harvard Medical School, Boston, MA USA; 4 Department of Diagnostic and Interventional Radiology, University Hospital, Freiburg, Germany; 5 Department of Radiology, Brigham and Women’s Hospital, Dana-Farber Cancer Institute, Harvard Medical School, Boston, MA USA; 6 Department of Radiology and Nuclear Medicine, CARIM & GROW, Maastricht University, Maastricht, The Netherlands

**Keywords:** Three-dimensional imaging, Translational research

## Abstract

Although artificial intelligence algorithms are often developed and applied for narrow tasks, their implementation in other medical settings could help to improve patient care. Here we assess whether a deep-learning system for volumetric heart segmentation on computed tomography (CT) scans developed in cardiovascular radiology can optimize treatment planning in radiation oncology. The system was trained using multi-center data (*n* = 858) with manual heart segmentations provided by cardiovascular radiologists. Validation of the system was performed in an independent real-world dataset of 5677 breast cancer patients treated with radiation therapy at the Dana-Farber/Brigham and Women’s Cancer Center between 2008–2018. In a subset of 20 patients, the performance of the system was compared to eight radiation oncology experts by assessing segmentation time, agreement between experts, and accuracy with and without deep-learning assistance. To compare the performance to segmentations used in the clinic, concordance and failures (defined as Dice < 0.85) of the system were evaluated in the entire dataset. The system was successfully applied without retraining. With deep-learning assistance, segmentation time significantly decreased (4.0 min [IQR 3.1–5.0] vs. 2.0 min [IQR 1.3–3.5]; *p* < 0.001), and agreement increased (Dice 0.95 [IQR = 0.02]; vs. 0.97 [IQR = 0.02], *p* < 0.001). Expert accuracy was similar with and without deep-learning assistance (Dice 0.92 [IQR = 0.02] vs. 0.92 [IQR = 0.02]; *p* = 0.48), and not significantly different from deep-learning-only segmentations (Dice 0.92 [IQR = 0.02]; *p* ≥ 0.1). In comparison to real-world data, the system showed high concordance (Dice 0.89 [IQR = 0.06]) across 5677 patients and a significantly lower failure rate (*p* < 0.001). These results suggest that deep-learning algorithms can successfully be applied across medical specialties and improve clinical care beyond the original field of interest.

## Introduction

Medical knowledge is increasing exponentially with an estimated doubling every few months as of 2020^1^. While this has improved healthcare across the world^2^, it is paralleled by increasingly specialized expert knowledge, which may be disproportionately distributed to high-resource medical centers, thus increasing healthcare disparities^3^. Recent advances in artificial intelligence (AI), and deep learning in particular, offer a novel way to improve and automate complex tasks that up until now could only be performed by professionals^4^. Typically, deep-learning applications are developed using labeled data generated by medical experts for domain-specific problems. As a result, this expert knowledge is encapsulated in the deep-learning system, providing the opportunity to disseminate this highly skilled expertise across medical domains, institutions and countries, with the potential to optimize patient care and reducing knowledge and economic disparities in undersupplied settings.

One area that could benefit from this concept are imaging-related specialties, such as radiology and radiation oncology. While the former uses imaging studies primarily for diagnosis, the latter relies on the same information for organ and tumor targeting, treatment planning and delivery, and monitoring. An integral part of radiotherapy treatment planning is segmenting organs at risk in the radiation field on computed tomography (CT) scans^5^. If appropriate resources are available, this is done manually by trained experts who require considerable time and are prone to inter- and intra-observer variability. If time or knowledge are limited, this crucial step to ensure treatment quality and patient safety may be neglected. Therefore, automating and optimizing this process of organ at risk segmentation by deep learning could improve clinical care at high speed and low additional cost, especially in underprivileged healthcare settings^6^.

Depending on the region of interest, different organs of varying complexity need to be segmented. Among those, the heart is of special interest as it is known that increasing radiation dose exposure to the organ is associated with future cardiac adverse events, such as coronary artery disease and heart failure^7,8^. Given their training, the highest anatomic expertise in cardiac imaging is likely found among cardiovascular radiologists, who focus on the diagnosis and monitoring cardiac-related diseases using dedicated image acquisition, reconstruction, and analysis techniques. Hence, disseminating this highly specific but narrow expert knowledge across medical domains and to institutions or countries with limited resources may enable more accurate treatment planning and measurement of cardiac radiation dose to optimize cardioprotective strategies in radiation oncology. This is of particular interest for patients with breast cancer as the heart and its substructures are in close proximity to the target area. Thus, reducing heart dose is of great importance to not harm the generally favorable outcomes of these patients.

**Fig. 1:**
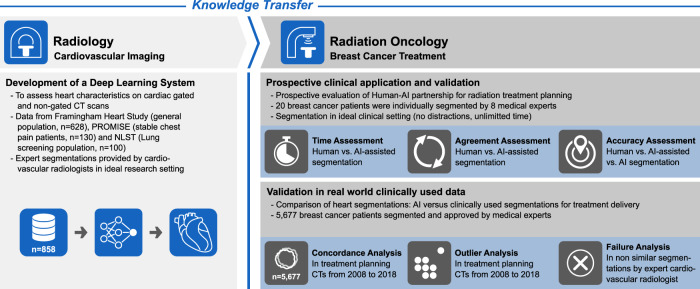
Study overview. A 3D deep-learning system was developed in cardiovascular radiology using CT scans from distinct and well-established cohorts. For training, medical experts segmented the heart in cardiac gated and non-gated CT scans. This specialized knowledge embedded in the deep-learning system was then transferred to radiation oncology and used to support treatment planning in patients with breast cancer.

Here, we investigate whether a deep-learning system developed in cardiovascular radiology can be applied for radiation oncology treatment planning. The deep learning system was developed for whole heart segmentation on input data provided by expert cardiovascular radiologists using dedicated, cardiac CT scans (see Fig. 1). We then applied this system in an independent dataset with real-world segmentations of 5677 patients with breast cancer to compare its performance to radiation oncology experts as well as to heart segmentations used in the clinic for treatment delivery. This study may serve as proof of principle to repurpose and leverage AI applications for optimizing patient care and reduce healthcare disparities across specialties, institutions, and countries.

## Results

### Training, tuning, and testing the deep-learning system

We trained and tuned the deep-learning system with 757 ECG-gated cardiac CTs as well as 100 low-dose chest screening CTs. The performance was tested in 1010 ECG-gated cardiac CTs and 296 low-dose chest screening CTs. Manual segmentations were done under the supervision of cardiovascular radiologists at the Massachusetts General Hospital. The deep learning system achieved a median Dice of 0.95 (IQR = 0.008) on the testing data.

### Prospective validation of AI assistance in clinical setting

**Fig. 2:**
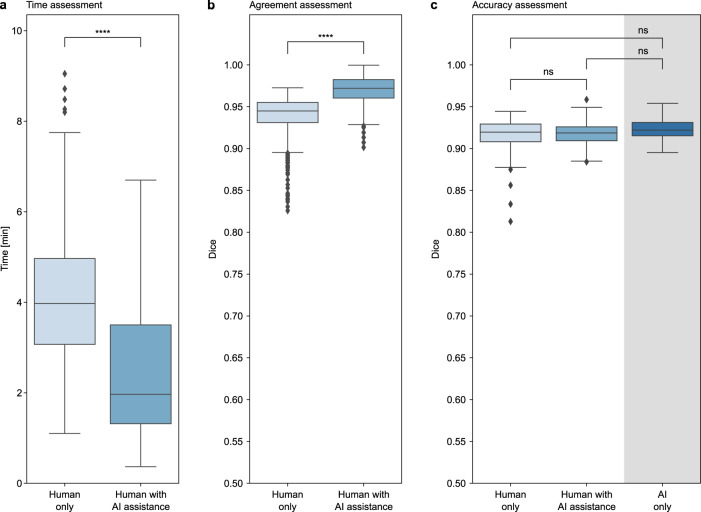
Comparison of human only, AI-assisted and AI-only segmentation. In a prospective assessment, 8 radiation oncology experts individually segmented the heart in 20 breast cancer treatment CTs. In a subsequent session, the same patients were segmented again with AI assistance. **a** The analysis shows that AI-assisted segmentation significantly reduces the time needed, (**b**) and agreement between medical experts significantly increases. **c** Comparing the manual-only, AI-assisted and AI-only segmentations to the reference segmentations of a radiation oncology expert with several years of experience shows no significant differences in accuracy. Each box represents the interquartile range (IQR, 25th and 75th percentiles) and the centerline the median of the results. The whiskers represent minimum and maximum data points, excluding outliers. Outliers are defined as greater than the 75th percentile +1.5×IQR and smaller than the 25th percentile −1.5×IQR and are denoted as diamonds.

To evaluate the deep-learning system for a clinical radiation oncology implementation, we compared the time needed to generate a clinically acceptable segmentation without and with the assistance of the deep learning system, and found a significant reduction by 50% (median 4.0 min [IQR 3.1–5.0] vs. 2.0 min [IQR 1.3–3.5]; *p* < 0.001) for the deep-learning-assisted approach compared to the current manual clinical workflow (Fig. 2a). At the same time, agreement of the segmentations significantly increased from a median Dice of 0.95 (IQR = 0.02) for the manual segmentations to 0.97 (IQR = 0.02) for the deep-learning-assisted approach (*p* < 0.001) (Fig. 2b). Along with the changes in time and variation, accuracy analysis revealed no significant differences between the manual and deep learning-assisted segmentations (median Dice 0.92 [IQR = 0.02] and 0.92 [IQR = 0.02], respectively; *p* = 0.50). Also, no significant differences were found between the deep-learning-only segmentations (median Dice 0.92 [IQR = 0.02]) and the manual as well as deep-learning-assisted approach (*p* = 0.2 and *p* = 0.10, respectively) (Fig. 2c). Additional results are provided in Supplementary Fig. 2.

### Validation of performance in real-world, clinically used data

**Fig. 3:**
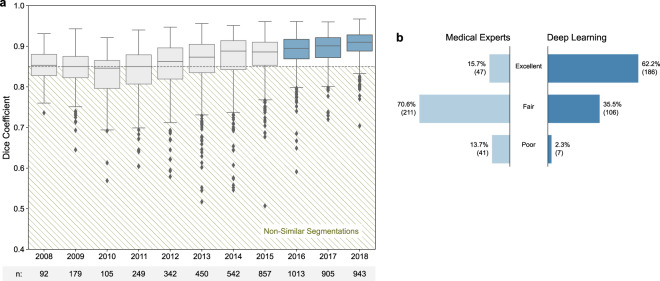
Similarity of manually and automatically generated segmentations. **a** Dice coefficient between the AI framework and clinically approved heart segmentations in 5677 scans acquired between 2008–2018. Non-similar segmentations with a dice coefficient below 0.85 (dashed line) were defined as failures. The boxes represent the interquartile range (IQR, 25th and 75th percentiles) and the centerlines the median of the results. The whiskers represent minimum and maximum data points, excluding outliers. Outliers are defined as greater than the 75th percentile + 1.5×IQR and smaller than the 25th percentile − 1.5×IQR and are denoted as diamonds. **b** Results of qualitative segmentation accuracy assessment in cases defined as failures between 2016–2018 (*n* = 299) by an expert cardiovascular radiologist. The results show significantly higher segmentation accuracy for AI as compared to radiation oncology experts (*p*-value < 0.0001).

In the subsequent assessment of the deep-learning system in real-world data used in clinical practice, the automated whole heart segmentations showed a high concordance (median Dice of 0.89 [IQR = 0.06]) with the clinically used segmentations across the entire cohort of 5,677 breast cancer patients. In a per-year analysis, the median Dice increased significantly from 0.85 (IQR = 0.05) in 2008 to 0.91 (IQR = 0.04) in 2018 (*p* < 0.001). In parallel, the percentage of failure cases with a Dice below 0.85 decreased from 46.7% to 5.6%. An overview is given in Fig. 3a.

**Fig. 4:**
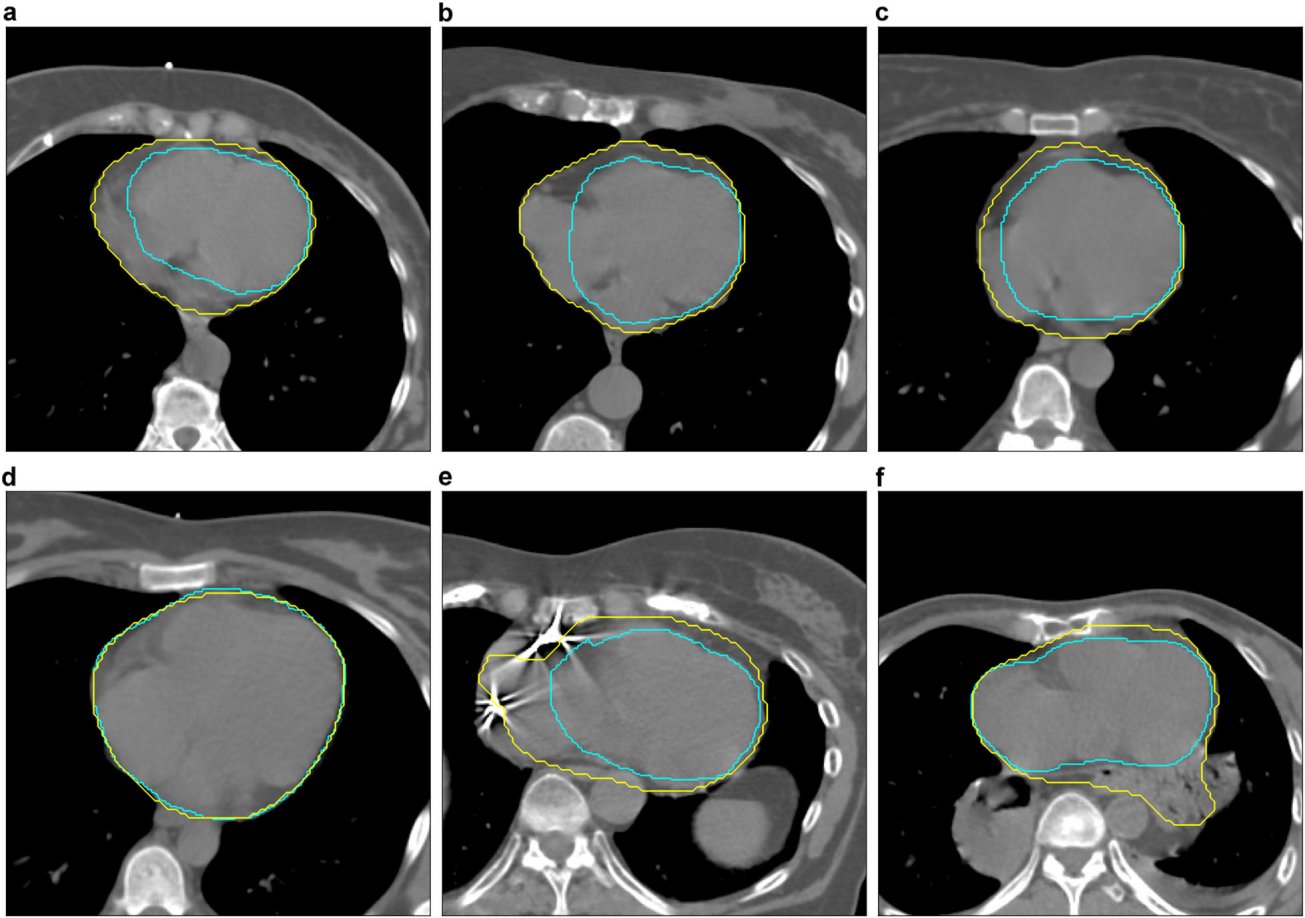
Segmentation accuracy of AI (Yellow) and manual (Cyan) segmentations. All manual segmentations were created by medical experts and approved by a radiation oncologist for treatment. Quality ratings (poor, fair, excellent) were made by a board-certified radiologist trained in cardiovascular imaging in a blinded pairwise fashion following the RADCOMP Breast Cancer Atlas. In (**a**–**c**), AI was rated excellent whereas the clinically used segmentations revealed a poor accuracy (Dice: 0.811, 0.826, and 0.826 respectively). **d** depicts an example with excellent segmentation accuracy for AI and radiation oncology experts (Dice: 0.960). **e** shows poor accuracy for both, AI and radiation oncology experts (Dice: 0.773). In (**f**), segmentation accuracy was rated poor for AI and fair for the radiation oncology expert segmentation (Dice: 0.833).

The detailed failure analysis of cases with a Dice below 0.85 in the subset of patients treated between 2016 and 2018 comprised 299 patients. The ratings performed by the cardiovascular radiologist revealed a significantly higher segmentation accuracy for the deep-learning system as compared to the manual, clinically used segmentations (*p* < 0.001). While the majority of deep-learning segmentation were rated as excellent (62.2% vs. 15.7% for the clinical segmentations), most of the historical clinically utilized segmentations were found to be of fair quality (70.6% vs. 35.5% for the deep-learning system). Poor accuracy was found for 13.7% of the clinical segmentations vs. 2.3% for the deep-learning approach (Fig. 3b**)**. Representative image examples are provided in Fig. 4.

## Discussion

In this study, we demonstrate that expert knowledge encapsulated in a deep-learning system can be disseminated across medical domains to help optimize the treatment of patients with breast cancer in radiation oncology. The dissemination of domain-specific expert knowledge across disciplines in medicine has profound clinical implications. With the rapid and ongoing growth of knowledge across all medical specialties, no single discipline or individual can master the entire field of medicine beyond their expertise^1^. On the contrary, continued sub-specialization and longer years of training lead to more narrow but highly skilled experts for particular fields or diseases. While this is beneficial if an expert is available onsite in resource-rich healthcare settings, the best possible care might not be deliverable to patients in low-resource areas^9^. In this context, expert knowledge encapsulated in deep-learning systems developed and tested for specific tasks, but then repurposed for different but related tasks in another specialty, institute or country, might be helpful to reduce knowledge and economic disparities, especially in undersupplied settings where such tasks might be neglected due to limited time or training. In such situations, an AI-mediated knowledge dissemination can create opportunities for human-AI partnerships to improve quality and safety in healthcare. Additionally, this approach maximizes the potential benefit of each expert annotated case, a particularly valuable aspect as deep-learning tasks depend on such annotated data, and the current paucity of these data limits deep-learning applications in medicine.

In our prospective clinical assessment, we evaluated the potential of a human-AI partnership for heart segmentation as part of breast cancer radiation treatment planning. In a previously published study Tschandl et al.^10^ showed how the human-AI relationship can improve image-based skin cancer diagnosis. In our study, we found that the partnership between dosimetrist and AI facilitated the generation of highly accurate heart segmentations in a significantly shorter time and with a significantly higher concordance compared to the current clinical standard in a high-resource medical center. At the same time, no differences in accuracy were observed. This is of considerable importance, as it helps to reduce labor-intensive manual work and could optimize quality while maintaining similar treatment standards^11^. Moreover, this is also an opportunity to improve the quality of care by reducing intra-reader and inter-reader variability both in radiology and radiation oncology^12,13^, which persist despite standardized guidelines have been proposed to ensure quality control^14^. Most interestingly, when comparing the manual and deep-learning-assisted segmentations to the deep-learning-only segmentations, no differences in accuracy were found. This suggests that human input might not be necessary at all to generate segmentations of similar quality as the current clinical standard, thus suggesting the beginning of a paradigm shift in segmentation for radiotherapy treatment planning and the potential to implement this technique in undersupplied hospitals, in which organ at risk segmentation is not performed due to limited resources.

These results were emphasized in our assessment of the deep-learning system in real-world, clinically used data of 5677 patients with breast cancer. Here, we could show a robust performance of the system without prior retraining. Although the median Dice was already high (0.85) in the subpopulation of patients treated in 2008, it significantly increased over the next decade to a median of 0.91. At the same time, the variance and number of patients with a Dice below 0.85 decreased. This is likely due to increased standardization of heart segmentations based on the 2016 RADCOMP guidelines, and recognition that heart dosimetry was intricately linked to radiotherapy toxicity and clinical outcomes^15^. In addition, it is of particular interest to gain a better understanding of failures before the potential implementation of a new deep-learning system into clinical workflows. In our analysis of outlier cases with a Dice below 0.85, we found a significantly higher failure rate in the clinically used segmentations as compared to the deep learning system (13.7% vs. 2.3%). This finding indicates that the current error rate in daily clinical practice could be significantly reduced by implementing the deep learning system for this heart segmentation task in radiotherapy planning. In addition, this may have implications for radiotherapy quality control by optimizing planning in order to minimize toxicity and enhance the therapeutic ratio. Moreover, creating a human-AI partnership for routine but clinically relevant tasks such as organ segmentation has the potential to fundamentally change and optimize clinical workflows^16^: (1) in high-resource centers by altering the role of medical experts from professionals spending substantial portions of their time manually generating segmentations to providing oversight of AI and quality control, while freeing up more time for higher value responsibilities such as face-to-face interactions with patients and/or complex clinical decision-making and (2) in low-resource settings by introducing new treatment possibilities that are currently neglected but paramount for patient safety and quality of care.

The robust performance of the deep-learning system is not only interesting from a clinical perspective, but also from a technical perspective^17^. In detail, we trained the deep-learning system using images and segmentations from cardiovascular radiology and assessed the possibility to transfer this learned knowledge to radiation oncology and further studied the human-AI partnership. The main difference between images of the radiology training cohorts and images of the oncology testing cohorts was that the training cohorts included mostly cardiac ECG-gated CTs acquired during a breathhold interval to reduce cardiac and respiratory motion artifacts while the testing cohorts consisted solely of non-gated scans and many of them acquired during free-breathing. Segmentations in non-gated scans are typically less accurate due to motion artifacts. This applies to both manual and automatic segmentations and explains the small performance drop of our network from the training cohorts to our testing cohorts. In addition, acquisition and reconstruction protocols as well as scanners varied widely, however, that did not seem to have a major impact on performance. The difference between images from the training and testing set are shown in an example in Supplementary Fig. 3, indicating the different image acquisition and reconstruction techniques used in radiology and radiation oncology, respectively.

Although the input data in the current study was considerably different from the data used for development, no systematic failures were observed and differences in acquisition and reconstruction protocols did not affect the segmentation performance. This indicates the robustness of the deep-learning system for potential applications in different clinical settings and beyond the primary intention of development. Additional data and patient baseline characteristics can be found in Supplementary Table 1.

There are limitations to our study that need to be addressed. Time needed for segmenting the heart was self-recorded by the medical expert, which makes inaccurate measurements more likely than if they were taken by an independent person. Moreover, with the investigated AI system, only whole heart segmentations are possible although there is increasing evidence suggesting that cardiac substructures are of more importance and more closely linked to outcome and cardiac toxicity. Also, as the primary focus of this study was on deep-learning-based expertise dissemination in a general setting, the analyses are lacking dose calculations and dedicated evaluations of treatment plans. In addition, as of now, only patients examined in the supine position can be analyzed by the deep-learning system given the input data used for training.

In conclusion, we demonstrated that expert knowledge encapsulated in a deep-learning system can be disseminated across medical domains and institutes to optimize patient care beyond the intended narrow field of application. Furthermore, we demonstrated that the disseminated domain-specific expertise can be repurposed to substantially optimize the efficiency and quality of care in the investigated example of heart segmentation for breast cancer radiotherapy planning.

## Methods

### Study design and population

An overview of the study design is given in Fig. 1. A search of the radiation oncology treatment planning system identified all breast cancer patients treated with radiotherapy in our institution’s Department of Radiation Oncology between 2004–2018 (*n* = 6751). Exclusion criteria were: corrupted imaging data (*n* = 380), missing/corrupted whole heart segmentations (*n* = 499) and images of patients other than in supine position (*n* = 195) resulting in a final study cohort of 5677 patients (Supplementary Fig. 1). The study was conducted under a protocol approved by the Dana-Farber/Harvard Cancer Center institutional review board, which waved written informed consent. CT images for treatment planning were acquired following the institutional standards without administration of intravenous contrast agent and with and without breath holding. As the inclusion timeframe is over a decade, scanners as well as acquisition and reconstruction protocols varied widely, thus reducing the likelihood that the results are biased towards a single institution or a specific vendor, scanner, or imaging technique, respectively. After reconstruction, images were transferred to the treatment planning system (Varian Eclipse, Varian Medical Systems, Palo Alto, California). All treatment plans and whole heart segmentation were created by trained medical experts following internal institution standards, and were in line with national guidelines as they became publicly available starting in 2016 (e.g. RADCOMP Breast Cancer Atlas^15^). All heart segmentations were approved by an attending radiation oncologist for use in clinical treatment planning.

### Development of AI system and domain transfer of expertise from cardiovascular radiology to radiation oncology

We developed a deep-learning system, which is able to automatically localize and segment the heart from a given CT scan using expert knowledge from cardiovascular radiologists. Therefore the proposed system consists of two consecutive steps, each using a separate 3-dimensional deep-learning model of the U-Net^18^ architecture. In-depth details of the system architecture, development, and application can be found in the Supplementary Methods (Supplementary Methods 1).

### Prospective validation of AI assistance in radiation oncology

To prospectively investigate the potential of a human AI partnership, we assessed the performance of 8 trained medical experts (certified medical dosimetrists) responsible for radiation treatment planning by asking each expert to segment the whole heart using their typical clinical routine without and then with access to the deep-learning system output. Measures of interest were (1) segmentation time, (2) agreement of the segmentations, defined as agreement between medical experts in the same patient, and (3) their anatomical accuracy, as outlined in RADCOMP Breast Cancer Atlas. For this assessment, 20 breast cancer patients were randomly selected from subjects treated in 2018. To avoid bias and ensure that the selected cases mirror a representative subset of the entire cohort, we calculated the dice coefficient between the AI segmentations and the clinically used segmentations before we started the trial with the dosimetrists. The mean dice was 0.90 (Std: 0.04) and the minimum and maximum dices were 0.78 and 0.94 respectively. As the network’s performance was varying in the selected cases, we could assume that there was no bias in the selected subsample. Furthermore, we used the parametric Welch’s t-test and non-parametric Mann–Whitney U test to compare the Dice coefficients of the subset and the full cohort. Both tests resulted in statistically not different dice coefficients (*p* = 0.293 and *p* = 0.153 respectively).

In a first segmentation session without distractions and no time limit, the medical experts were asked to segment the heart using the technique they would use in routine clinical care and recorded the time needed per patient. In a subsequent segmentation session 2 weeks later, the heart of the same 20 patients was pre-segmented with the deep-learning system prior to the start of the session. The 8 medical experts were then asked to review and, where necessary, modify the deep-learning segmentations until they were clinically acceptable for radiotherapy planning. Again, there were no other restrictions made and the time needed to modify the segmentations was self-recorded by each medical expert. The segmentations of a senior radiation oncologist with more than 16 years of experience in breast cancer treatment acquired in the same setting were used as reference standard for the medical experts as well as for the deep-learning segmentations.

### Validation in real-world data used for radiation treatment delivery

To investigate the application and robustness of the deep-learning system in real-world clinically used data, we analyzed its performance across the entire study cohort using the historical, clinically used segmentations as comparators. Based on a subjective review, a Dice ≥0.85 was arbitrarily defined as “similar segmentation”. For quality control and to generate a better understanding for reasons of discordance between deep-learning and clinically utilized heart segmentations, we manually analyzed cases considered as failures (Dice < 0.85) in a subset of patients treated between 2016–2018 (*n* = 299). This timeframe was chosen to explore failure rates in the most recently treated patients following the latest implemented guideline update^15^. A board-certified radiologist trained in cardiovascular imaging with 6 years of experience rated anatomical accuracy of the historical, manually created and clinically used as well as the deep-learning segmentations on a 3-point Likert scale (1 = poor, 2 = fair, 3 = excellent). The reading session was performed in a pairwise fashion and blinded to the segmentation technique used.

### Statistical analysis

All statistical analyses were performed in Python (V2.7). Data are presented as median and interquartile ranges (IQR). Similarity of manual and deep-learning segmentations was measured using the Dice coefficient^19,20^ with a smoothing factor of one. Furthermore, we calculated the symmetric surface distance and Hausdorff distance using the MedPy Python package (V0.4.4). For pairwise comparison a non-parametric Wilcoxon signed-rank test was performed due to violation of the normality assumption. To perform the parametric Welch’s t-test and non-parametric Mann-Whitney U test we used the SciPy.stats Python package (V1.2.3). All p-values were two-sided and considered statistically significant below 0.05.

### Reporting summary

Further information on research design is available in the Nature Research Reporting Summary linked to this article.

## Supplementary information

Supplementary Information

Reporting Summary

## Data Availability

The trained models are shared under the GNU General Public License v3.0^21^ at our webpage https://aim.hms.harvard.edu/DeepHeartRO. Due to privacy agreements with our institutions we can not share CT imaging or segmentation data. For that reason we provide test data from a publicly available dataset with automatic heart segmentations.

## Code availability

The full code of the deep-learning system and statistical analysis is shared under the GNU General Public License v3.0^21^ at https://aim.hms.harvard.edu/DeepHeartRO.
