# Supplementary material for: Deep-learning system to improve the quality and efficiency of volumetric heart segmentation for breast cancer
Source: NPJ Digit Med. 2021 Mar 5;4:43. doi: 10.1038/s41746-021-00416-5 (PMC7935874; doi:10.1038/s41746-021-00416-5)
Supplement: Supplementary file 1 — Supplementary Information [file 41746_2021_416_MOESM1_ESM.pdf]

Supplementary Information to:

Deep-learning system to improve the quality and efficiency of volumetric heart segmentation for breast cancer

Roman Zeleznik; Jakob Weiss; Jana Taron; Christian Guthier; Danielle S. Bitterman; Cindy Hancox; Benjamin H. Kann; Daniel W. Kim; Rinaa S. Punglia; Jeremy Bredfeldt; Borek Foldyna; Parastou Eslami; Michael T. Lu; Udo Hoffmann; Raymond Mak; Hugo J.W.L. Aerts

I Supplementary Methods

- **Supplementary Methods 1.** Development of the AI system

II Supplementary Figures

- **Supplementary Figure 1.** Consort diagram.
- **Supplementary Figure 2.** Comparison of human only, AI-assisted and AI only segmentation using the average symmetric surface distance.
- **Supplementary Figure 3.** Comparison of training and testing CT scans.

III Supplementary Tables

- **Supplementary Table 1:** Data and patient baseline characteristics

IV Supplementary References

I Supplementary Methods

Methods 1. Development of the AI system

We developed a deep learning system, which is able to automatically segment the heart from a given CT scan using expert knowledge from cardiovascular radiologists. The main purpose for the development of this tool was to subsequently quantify cardiac phenotypes such as heart volume, epicardial fat, and coronary artery calcium as predictors for future cardiac events[1].

The majority of the training data included non-contrast ECG-gated cardiac CT scans from the Framingham Heart Study Offspring[2] and Third Generation cohort participants (FHS-CT1, n=129) taken between 2002 and 2005 as well as scans from the second examination cycle of the Third Generation Cohort, taken between 2008 and 2011 (FHS-CT2, n=499)[2,3]. Furthermore we included participants from the Prospective Multicenter Imaging Study for Evaluation of Chest Pain (PROMISE, n=130)[4,5] as well as non ECG-gated low-dose chest CT for lung cancer screening from the National Lung Screening Trial (NLST, n=100)[6]. The heart segmentation in the training cohort was done manually under the supervision of cardiovascular radiologists at the Massachusetts General Hospital on dedicated workstations using 3D Slicer (V4)[7].

In total we trained and tuned the deep learning system with 858 CTs and tested its performance in 1010 ECG-gated cardiac CTs and 296 low dose chest screening CTs. To rigorously assess the generalizability of the deep learning system we maximized the sample size in the testing set, and hence, kept the training set small. An initial run with a training set approximately a third of the final training set led to good average performance but with many outliers. Extending the training set to its final size increased the average performance slightly, and more importantly the number of outliers was reduced significantly.

The proposed deep learning system consists of two consecutive steps to localize and subsequently segment the heart using a separate 3-dimensional deep learning model of the U-Net[8] architecture for each of the two steps. The first step was necessary to reliably localize the heart in CT scans with differences in size, resolution, area captured and field of view, depending on the cohort, scanner used, and site acquiring the scan. This localization step was performed by segmenting the heart in heavily downsampled scans. Afterwards, the output of the network was up-sampled resulting in a very rough heart segmentation which we used for placing a bounding box for the subsequent high resolution segmentation step.

For training the localization network the scans were cropped and down-sampled to a size of $112 \times 112 \times 112$ voxels (vx) and a resolution of 3mm/vx in all directions, to fit into the memory of the Graphics Processing Unit (GPU). The whole cohort was split 70/30% for training and tuning the network and training took 1,200 epochs. To increase the

training data we augmented the scans by applying translations within ±10vx in the axial plane for heart localization and rotations of ±4 degrees around the sagittal, transversal and longitudinal axis.

In a second step a deep learning network was trained to segment the heart with high resolution. Using the rough heart segmentation from step one, the scans were cropped to 384×384×80vx cubes around the heart center and again down-sampled to 128×128×80vx (2.0×2.0×2.5mm/vx resolution) to fit into the GPU memory. The cohort was split 70/30% for training and tuning and the data was augmented by applying rotations of ±35 degrees around the sagittal axis and translations of ±20vx in the axial plane. After training and tuning the network to a satisfying performance we trained the network again over 1,000 epochs using the full cohort. The output of the network was up-sampled to initial CT scan size leading to an accurate heart segmentation.

Training, tuning and testing was done on a Linux workstation using Tensorflow-GPU (V1.14) and Keras (V2.3.1) with NVIDIA CUDA (V10.2). The only notable hardware requirement was to have at least 64 gigabyte of GPU memory to fit a reasonable batch-size of input volumes for the heart segmentations.

To investigate the proposed concept of transferring deep learning encapsulated knowledge across medical specialties, we applied the deep learning system onto the planning CT scans from radiation oncology to produce whole heart segmentations in the 5,677 breast cancer patients without any retraining. Additional patient and image characteristics as well as acquisition and reconstruction parameters can be found in the **Supplementary Table 1**.

II Supplementary Figures

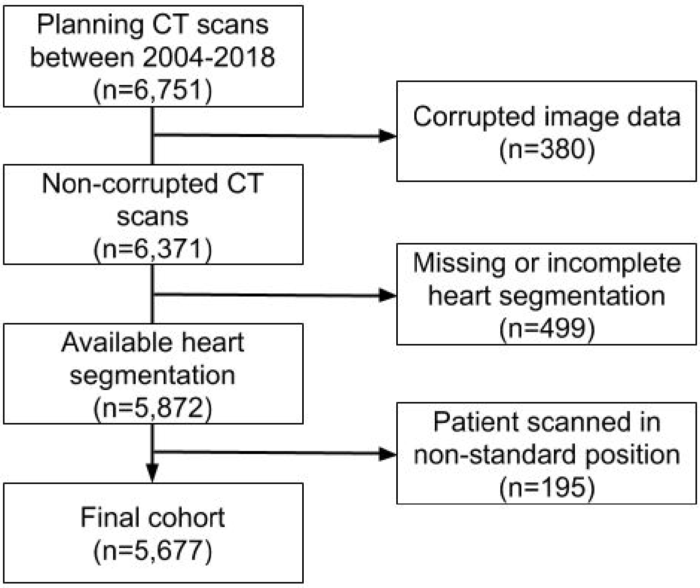

Supplementary Figure 1. Consort diagram.

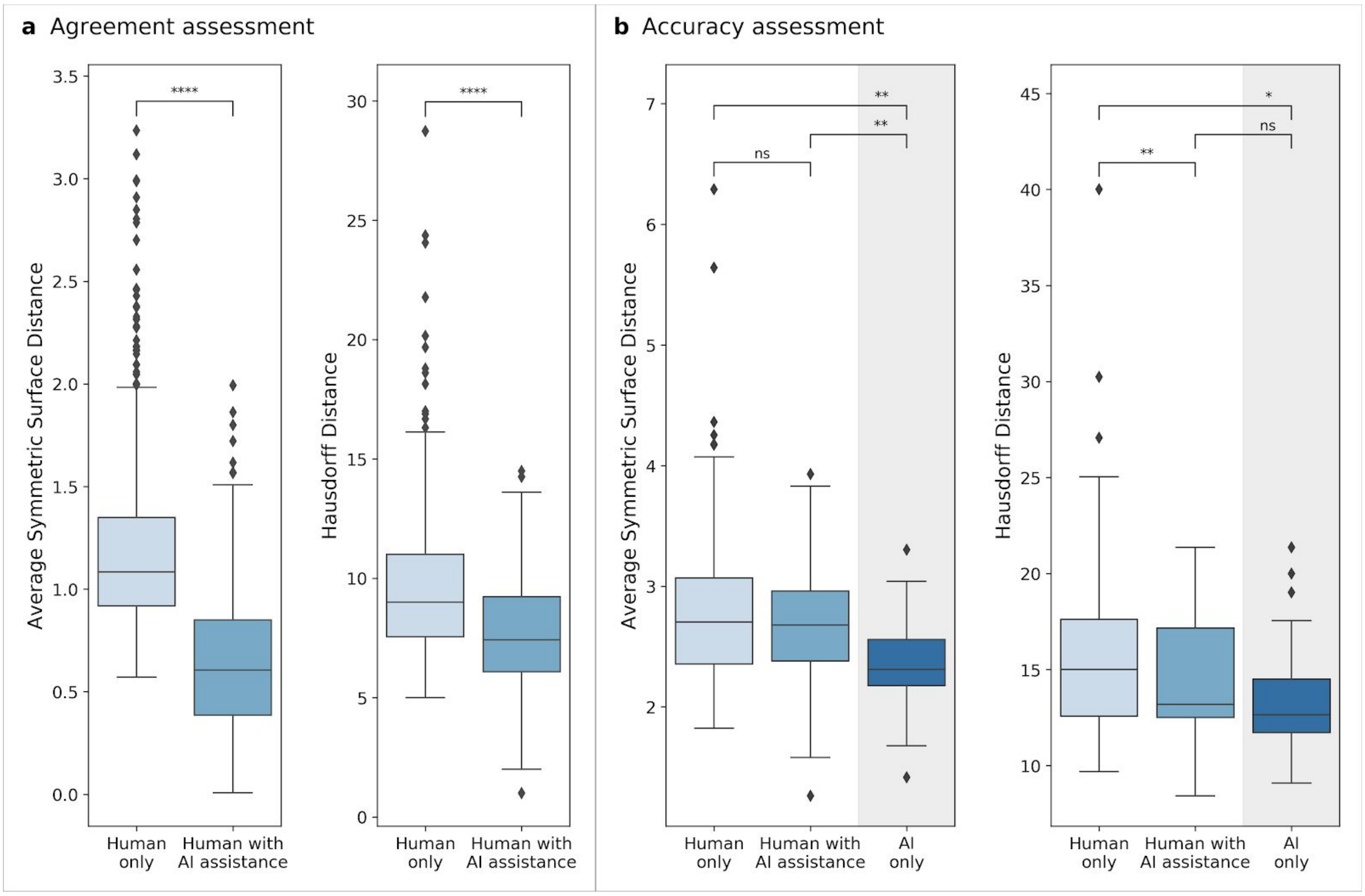

Supplementary Figure 2. Comparison of human only, AI-assisted and AI only segmentation. In a prospective assessment, 8 radiation oncology experts individually segmented the heart in 20 breast cancer treatment CTs. In a subsequent session, the same patients were segmented again with AI assistance. **a**, Assessment of the segmentation agreement between medical experts without and with AI-assistance, using the average symmetric surface distance (left) and the Hausdorff distance (right). **b**, Comparing the Human-only and AI-assisted and AI-only segmentations to the reference segmentations of a radiation oncology expert with several years of experience, using the average symmetric surface distance (left) and the Hausdorff distance (right). Each box represents the interquartile range (IQR, 25th and 75th percentiles) and the centerline the median of the results. The whiskers represent minimum and maximum data points, excluding outliers. Outliers are defined as greater than the 75th percentile + 1.5 $\times$ IQR and smaller than the 25th percentile - 1.5 $\times$ IQR and are denoted as diamonds.

a b

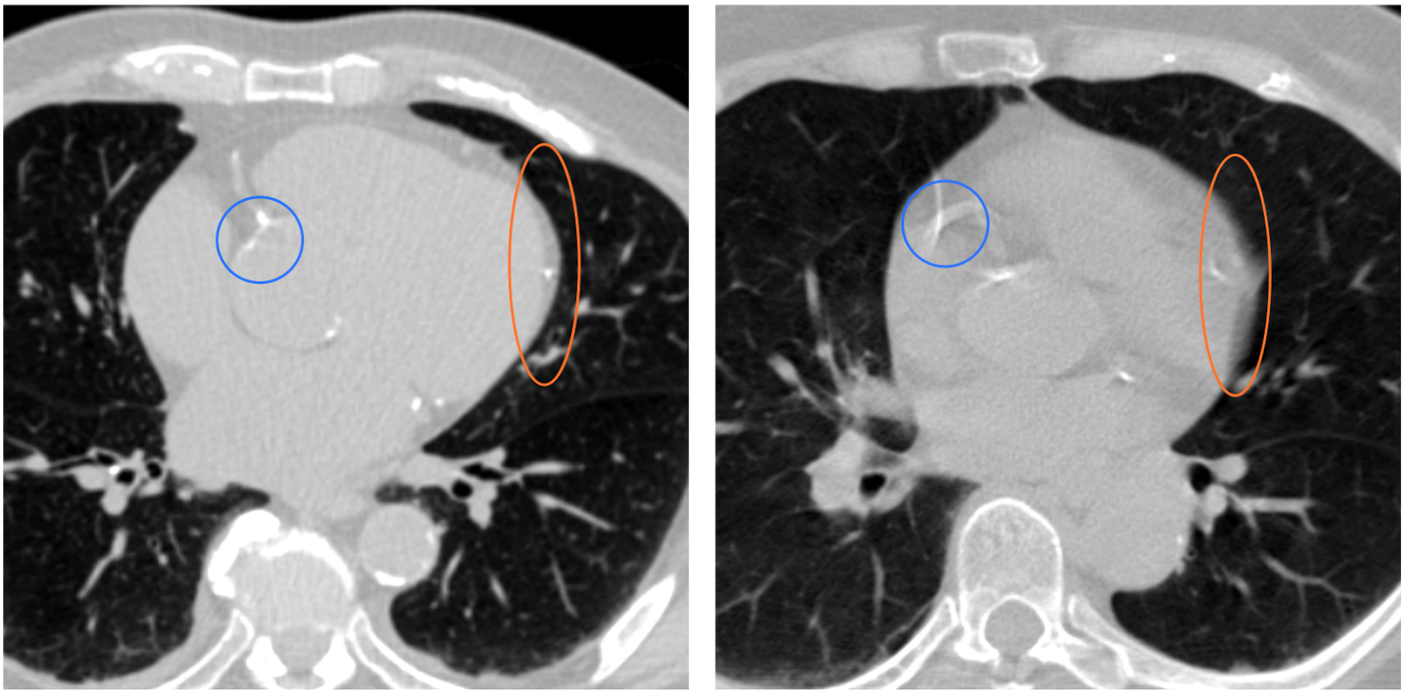

Supplementary Figure 3. Comparison of training and testing CT scans. While the training data consisted mainly of dedicated cardiac CT scans acquired with ECG-gating and during breath-hold (a), the testing data included non-gated scans only (b). Non-gated scans have more motion blurring (orange circle) as well as motion artifacts (blue circle).

III Supplementary Tables

<table>
<tr><th colspan="7">Supplementary Table 1: Data and patient baseline characteristics</th></tr>
<tr><th></th><th colspan="3">Training data (n=858)</th><th colspan="3">Study data (n=5,677)</th></tr>
<tr><td colspan="7">Patient Characteristics</td></tr>
<tr><td>Gender (% female)</td><td colspan="3">42.9</td><td colspan="3">99.6</td></tr>
<tr><td>Age: mean (std)</td><td colspan="3">61.2 (11.4)</td><td colspan="3">58.2 (11,7)</td></tr>
<tr><td colspan="7">Image Characteristics</td></tr>
<tr><td>Image Size [px]</td><td>512</td><td>512</td><td>Mean: 78 (Std: 31.9)
Min: 38; Max: 345</td><td>512</td><td>512</td><td>Mean: 138.5 (Std: 13.9)
Min: 100; Max: 230</td></tr>
<tr><td>Resolution [mm/px]</td><td colspan="2">Mean: 0.67 (Std: 0.04)
Min: 0.55, Max: 0.68</td><td>2.0: 12%; 2.5: 88%</td><td colspan="2">Mean: 1.1 (Std: 0.15)
Min: 0.7; Max:1.6</td><td>2.5: 95%; 3.0: 7%</td></tr>
<tr><td colspan="7">Acquisition and Reconstruction Characteristics</td></tr>
<tr><td>Peak Kilovoltage [kV]</td><td colspan="3">100-140</td><td colspan="3">120-140</td></tr>
<tr><td>Max X-Ray tube current: mean, [mA]</td><td colspan="3">300.4 (Std: 111.2; Min:38; Max: 1142)</td><td colspan="3">339.3 (Std:93.2; Min: 64 Max: 658)</td></tr>
<tr><td>Exposure time: mean, [ms]</td><td colspan="3">322.1 (Std: 152.8; Min:168; Max:1140)</td><td colspan="3">1532.5 (Std: 639.7; Min: 500; Max: 2851)</td></tr>
<tr><td>Reconstruction diameter: mean, [mm]</td><td colspan="3">317.0 (59.8; Max:134; Min: 500)</td><td colspan="3">559.2 (Std: 76.7; Min: 300; Max: 800)</td></tr>
<tr><td>Convolution kernel [%]</td><td colspan="3">B: 1.4; B20f: 0.3; B25f: 0.5; B30f: 4.4; B31s: 0.3; B35f: 4.9; B36f: 0.3; B50f: 1.0; BONE: 0.5; C: 0.9; CB: 1.6; FC10: 0.3; FC12: 2.3; FC51: 0.8; I30f2: 0.3; I30f3: 0.4; STANDARD: 78.4; ‘71’: 0.4; None: 1.0;</td><td colspan="3">STANDARD: 91.8, B: 6.6, B19s: 0.1, B46f: 0.1, SOFT: 0.1, n/a: 1.3</td></tr>
<tr><td>Manufacturer [%]</td><td colspan="3">GE MEDICAL SYSTEMS - LightSpeed: 44.4; GE MEDICAL SYSTEMS - Discovery: 34.6; GE MEDICAL SYSTEMS - HiSpeed: 0.3; Hitachi Medical Corporation - SCENARIA: 0.4; Philips – Brilliance: 1.7; Philips – iCT: 1.2; Philips - Ingenuity CT: 0.3; Philips - Mx8000: 0.9; SIEMENS - Somaris: 0.3; SIEMENS - Definition: 0.6; SIEMENS - Volume Zoom: 3.4; SIEMENS - Sensation: 4.2; SIEMENS - SOMATOM: 3.3; SIEMENS - Emotion: 0.3; TERARECON - Discovery: 1.0; TOSHIBA - Aquilion: 3.1;</td><td colspan="3">GE MEDICAL SYSTEMS - Discovery : 11.8; GE MEDICAL SYSTEMS - LIGHTSPEED : 77.9; Philips - Big Bore : 6.6; Varian Medical Systems : 3.7</td></tr>
<tr><td colspan="7">n/a indicates that the characteristic was not available for a patient</td></tr>
</table>

IV Supplementary References

1. Zeleznik, R. *et al.* Deep-Learning Quantification of Coronary Calcium on CT and Mortality in the National Lung Screening Trial (NLST). https://rsna2019.rsna.org/program/details/?publicid=RC303-02 (2019).
2. Kannel, W. B., Feinleib, M., McNamara, P. M., Garrison, R. J. & Castelli, W. P. An investigation of coronary heart disease in families. The Framingham offspring study. *Am. J. Epidemiol.* **110**, 281–290 (1979).
3. Splansky, G. L. *et al.* The Third Generation Cohort of the National Heart, Lung, and Blood Institute's Framingham Heart Study: Design, Recruitment, and Initial Examination. *Am. J. Epidemiol.* **165**, 1328–1335 (2007).
4. Douglas, P. S. *et al.* PROspective Multicenter Imaging Study for Evaluation of chest pain: rationale and design of the PROMISE trial. *Am. Heart J.* **167**, 796–803.e1 (2014).
5. Douglas, P. S. *et al.* Outcomes of anatomical versus functional testing for coronary artery disease. *N. Engl. J. Med.* **372**, 1291–1300 (2015).
6. National Lung Screening Trial Research Team *et al.* Reduced lung-cancer mortality with low-dose computed tomographic screening. *N. Engl. J. Med.* **365**, 395–409 (2011).
7. Pieper, S., Halle, M. & Kikinis, R. 3D Slicer. in *2004 2nd IEEE International Symposium on Biomedical Imaging: Nano to Macro (IEEE Cat No. 04EX821)* 632–635 Vol. 1 (2004).
8. Ronneberger, O., Fischer, P. & Brox, T. U-Net: Convolutional Networks for Biomedical Image Segmentation. *Lecture Notes in Computer Science* 234–241 (2015) doi:10.1007/978-3-319-24574-4_28.
