# Supplementary material for: Deep-learning system to improve the quality and efficiency of volumetric heart segmentation for breast cancer
Source: NPJ Digit Med. 2021 Mar 5;4:43. doi: 10.1038/s41746-021-00416-5 (PMC7935874; doi:10.1038/s41746-021-00416-5)
Supplement: Supplementary file 2 — Reporting Summary [file 41746_2021_416_MOESM2_ESM.pdf]

nature research

Corresponding author(s): Hugo Aerts, PhD
Phone: 617.525.7156
Email: haerts@bwh.harvard.edu

Last updated by author(s): Dec 29, 2020

Reporting Summary

Nature Research wishes to improve the reproducibility of the work that we publish. This form provides structure for consistency and transparency in reporting. For further information on Nature Research policies, see our Editorial Policies and the Editorial Policy Checklist.

Statistics

For all statistical analyses, confirm that the following items are present in the figure legend, table legend, main text, or Methods section.

n/a	Confirmed	
☐	☒	The exact sample size (n) for each experimental group/condition, given as a discrete number and unit of measurement
☒	☐	A statement on whether measurements were taken from distinct samples or whether the same sample was measured repeatedly
☐	☒	The statistical test(s) used AND whether they are one- or two-sided<br>*Only common tests should be described solely by name; describe more complex techniques in the Methods section.*
☒	☐	A description of all covariates tested
☒	☐	A description of any assumptions or corrections, such as tests of normality and adjustment for multiple comparisons
☐	☒	A full description of the statistical parameters including central tendency (e.g. means) or other basic estimates (e.g. regression coefficient) AND variation (e.g. standard deviation) or associated estimates of uncertainty (e.g. confidence intervals)
☐	☒	For null hypothesis testing, the test statistic (e.g. F, t, r) with confidence intervals, effect sizes, degrees of freedom and P value noted<br>*Give P values as exact values whenever suitable.*
☒	☐	For Bayesian analysis, information on the choice of priors and Markov chain Monte Carlo settings
☒	☐	For hierarchical and complex designs, identification of the appropriate level for tests and full reporting of outcomes
☒	☐	Estimates of effect sizes (e.g. Cohen's d, Pearson's r), indicating how they were calculated

Our web collection on statistics for biologists contains articles on many of the points above.

Software and code

Policy information about availability of computer code

Data collection	A search of the radiation oncology treatment planning system identified all breast cancer patients treated with radiotherapy in our institution's Department of Radiation Oncology between 2004-2018 (n=6,751). After reconstruction, images were transferred to the treatment planning system (Varian Eclipse, Varian Medical Systems, Palo Alto, California). All treatment plans and whole heart segmentation were created by trained medical experts following internal institution standards, and were in line with national guidelines as they became publicly-available.
Data analysis	Python V2.7.17; pip V20.2; Nvidia GPU driver V440.100; Nvidia Cuda 10.1; Nvidia cuDNN V7.6; R V3.6.3; RStudio V1.3.1073; 3D Slicer V4;<br>Python-packages: pydicom V1.4.2; SimpleITK V1.2.4, Numpy V1.16.6; Scipy V1.2.3; h5py V2.10.0; scikit-learn package V0.20.4; Scipy V1.2.3;<br>Tensorflow-GPU V1.15.0; Keras V2.3.1;<br>R-packages: ICC v2.3.0; Survcomp V1.36.1; Survminer V0.4.8; Survival V3.2.3

For manuscripts utilizing custom algorithms or software that are central to the research but not yet described in published literature, software must be made available to editors and reviewers. We strongly encourage code deposition in a community repository (e.g. GitHub). See the Nature Research guidelines for submitting code & software for further information.

Data

Policy information about availability of data

All manuscripts must include a data availability statement. This statement should provide the following information, where applicable:

- Accession codes, unique identifiers, or web links for publicly available datasets
- A list of figures that have associated raw data
- A description of any restrictions on data availability

The trained models are shared under the MIT license at our webpage https://aim.hms.harvard.edu/DeepHeartRO. Due to privacy agreements with our institutions we can not share CT imaging or segmentation data. For that reason we provide test data from a publicly available data set with automatic heart segmentations.

Field-specific reporting

Please select the one below that is the best fit for your research. If you are not sure, read the appropriate sections before making your selection.

☒ Life sciences ☐ Behavioural & social sciences ☐ Ecological, evolutionary & environmental sciences

For a reference copy of the document with all sections, see nature.com/documents/nr-reporting-summary-flat.pdf

Life sciences study design

All studies must disclose on these points even when the disclosure is negative.

Sample size	Development of the deep learning network: 2,164<br>Study population: 5,677
Data exclusions	Corrupted image data: 380<br>Missing or incomplete heart segmentation: 499<br>Patient scanned in non-standard position: 195
Replication	The results can be replicated entirely as the deep learning system produces the exact same results in every run.
Randomization	For the prospective validation 20 breast cancer patients were randomly selected from subjects treated in 2018. To avoid bias and ensure that the selected cases mirror a representative subset of the entire cohort, we calculated the dice coefficient between the AI segmentations and the clinically used segmentations before we started the trial with the dosimetrists. The mean dice was 0.90 (Std: 0.04) and the minimum and maximum dices were 0.78 and 0.94 respectively. As the network's performance was varying in the selected cases, we could assume that there was no bias in the selected subsample. Furthermore we used the parametric Welch's t-test and non-parametric Mann-Whitney U test to compare the Dice coefficients of the subset and the full cohort. Both tests resulted in statistically not different dice coefficients (p=0.293 and p=0.153 respectively).<br>For the validation in real world data the full study cohort was used.
Blinding	Blinding was not relevant as all medical experts segmented all patients.

Reporting for specific materials, systems and methods

We require information from authors about some types of materials, experimental systems and methods used in many studies. Here, indicate whether each material, system or method listed is relevant to your study. If you are not sure if a list item applies to your research, read the appropriate section before selecting a response.

Materials & experimental systems

n/a	Involved in the study	
☒	☐	Antibodies
☒	☐	Eukaryotic cell lines
☒	☐	Palaeontology and archaeology
☒	☐	Animals and other organisms
☐	☒	Human research participants
☒	☐	Clinical data
☒	☐	Dual use research of concern

Methods

n/a	Involved in the study	
☒	☐	ChIP-seq
☒	☐	Flow cytometry
☒	☐	MRI-based neuroimaging

Human research participants

Policy information about studies involving human research participants

Population characteristics	Study population: 99.6 % female; Mean age: 58.2 (STD: 11,7), Ethnicity: White: 81.9, Black: 4.9, Asian: 3.5, Latino: 0.8, Aboriginal: 0.4, American Indian: 0.1, Pacific Islander: 0.1, n/a: 8.3
Recruitment	We included all breast cancer patients treated with radiotherapy in our institution's Department of Radiation Oncology between 2004-2018.
Ethics oversight	The study was conducted under a protocol approved by the Dana-Farber/Harvard Cancer Center institutional review board.

Note that full information on the approval of the study protocol must also be provided in the manuscript.
